# Role of Oxidative Stress in Diabetic Cardiomyopathy

**DOI:** 10.3390/antiox11040784

**Published:** 2022-04-15

**Authors:** Bart De Geest, Mudit Mishra

**Affiliations:** 1 Centre for Molecular and Vascular Biology, Catholic University of Leuven, 3000 Leuven, Belgium; 2 Department of Cardiothoracic Surgery, University Medical Center Utrecht, 3584 CX Utrecht, The Netherlands; drmuditm@gmail.com

**Keywords:** diabetic cardiomyopathy, oxidative stress, type 2 diabetes mellitus, heart failure, HFpEF, HFrEF, primary antioxidant enzymes, apolipoprotein A-I, pathological hypertrophy, pathological remodeling

## Abstract

Type 2 diabetes is a redox disease. Oxidative stress and chronic inflammation induce a switch of metabolic homeostatic set points, leading to glucose intolerance. Several diabetes-specific mechanisms contribute to prominent oxidative distress in the heart, resulting in the development of diabetic cardiomyopathy. Mitochondrial overproduction of reactive oxygen species in diabetic subjects is not only caused by intracellular hyperglycemia in the microvasculature but is also the result of increased fatty oxidation and lipotoxicity in cardiomyocytes. Mitochondrial overproduction of superoxide anion radicals induces, via inhibition of glyceraldehyde 3-phosphate dehydrogenase, an increased polyol pathway flux, increased formation of advanced glycation end-products (AGE) and activation of the receptor for AGE (RAGE), activation of protein kinase C isoforms, and an increased hexosamine pathway flux. These pathways not only directly contribute to diabetic cardiomyopathy but are themselves a source of additional reactive oxygen species. Reactive oxygen species and oxidative distress lead to cell dysfunction and cellular injury not only via protein oxidation, lipid peroxidation, DNA damage, and oxidative changes in microRNAs but also via activation of stress-sensitive pathways and redox regulation. Investigations in animal models of diabetic cardiomyopathy have consistently demonstrated that increased expression of the primary antioxidant enzymes attenuates myocardial pathology and improves cardiac function.

## 1. General Introduction

A cardiomyopathy is defined by the European Society of Cardiology in 2008 as a myocardial disorder in which the heart muscle is structurally and functionally abnormal, in the absence of coronary artery disease, hypertension, valvular disease, and congenital heart disease sufficient to cause the observed myocardial abnormality [1]. Diabetic cardiomyopathy is a cardiomyopathy that fulfills this definition in subjects with diabetes mellitus. Major pathological features of diabetic cardiomyopathy are cardiomyocyte hypertrophy, myocardial interstitial and perivascular fibrosis, inflammation, apoptosis, and increased markers of oxidative stress [2].

In 1972, Rubler et al. described the postmortem findings and clinical records of 27 patients with proven diabetic glomerulosclerosis [3]. The authors aimed to identify patients with evidence of primary myocardial disease. Twenty-three patients were excluded because of the presence of hypertension, of significant obstruction of the major epicardial coronary arteries, or of valvular heart disease. Four patients demonstrated cardiomegaly and congestive heart failure of no known cause. These cases meet the criteria of the contemporary definition of cardiomyopathy of 2008 [1] and constitute the first description of diabetic cardiomyopathy. Pathological findings in these patients were cardiomyocyte hypertrophy, diffuse myocardial fibrosis, and abnormalities of the intramyocardial microvascular vessels. The phenotype of the patients described by Rubler et al. corresponds to a dilated cardiomyopathy [3]. However, the phenotype of most patients with diabetic cardiomyopathy resembles more that of a restrictive cardiomyopathy and typically occurs in subjects with obesity and type 2 diabetes mellitus [4]. These patients have thickened left ventricular walls, no dilatation of the left ventricular cavity or even shrinkage of the left ventricular cavity, a large left atrium, and elevated left ventricular filling pressures. These phenotypes are distinct phenotypes, and an evolution from the restrictive phenotype to the dilated phenotype appears to be unlikely or to occur infrequently [5]. These two phenotypes must reflect different pathophysiological mechanisms [6,7] that lead to left ventricular chamber dilatation in the dilated phenotype and a normal, quasi-normal, or small left ventricular chamber size in the restrictive phenotype [8]. The dilated phenotype mainly occurs in longstanding type 1 diabetes mellitus whereas the restrictive phenotype is predominantly observed in obese subjects with type 2 diabetes mellitus [5]. In the presence of symptoms, a diagnosis of heart failure with reduced ejection fraction (HFrEF) (ejection fraction ≤ 40%) or of heart failure with preserved ejection fraction (HFpEF) (ejection fraction ≥ 50%) is made.

Oxidative stress plays a key pathophysiological role in the initiation and progression of pathological hypertrophy and pathological remodeling, and in the development and evolution of heart failure both in subjects with diabetes and subjects without diabetes [9,10]. Pathological cardiac hypertrophy is by definition associated with cardiac dysfunction [11]. Microscopic features of pathological hypertrophy are cardiomyocyte hypertrophy associated with myocardial fibrosis and apoptosis in the myocardium [12]. These features are caused by oxidative stress. Oxidative stress increases the expression of transforming growth factor-β, stimulates transformation of fibroblasts to myofibroblasts, and promotes collagen synthesis, leading to cardiac fibrosis [13,14]. In the first stage, diabetic cardiomyopathy includes an asymptomatic subclinical period characterized by abnormalities of cardiac structure and cardiac function, including left ventricular hypertrophy and myocardial fibrosis, augmented myocardial stiffness, and subclinical diastolic dysfunction. In the symptomatic stage, these abnormalities may evolve to HFpEF, whereas in the presence of marked systolic dysfunction, HFrEF may develop [15,16,17,18].

Diabetes mellitus increases the risk of coronary heart disease in both sexes (hazard ratio 1.99 and 2.93 for men and women, respectively) [19]. This review focuses on diabetic cardiomyopathy. Consequently, heart disease secondary to coronary artery disease in diabetic subjects will not be considered. The risk of incident heart failure in the Framingham Heart Study was 2.4-fold higher in diabetic men and 5.0-fold higher in diabetic women independent of age, coronary disease, hypertension, and body mass index [20].

As will be discussed in Section 3, there are several diabetes-specific mechanisms that contribute to prominent oxidative distress in the diabetic heart. Since the prevalence of type 2 diabetes mellitus is more than 20-fold higher than the prevalence of type 1 diabetes, diabetic cardiomyopathy is mainly observed in type 2 diabetic patients. A concise discussion of the pathogenesis of type 2 diabetes mellitus and its relation to oxidative stress is, therefore, indicated to subsequently focus on the role of oxidative stress in diabetic cardiomyopathy in Section 3 and prevention and intervention studies in Section 4.

## 2. Oxidative Stress and Type 2 Diabetes Mellitus

### 2.1. Epidemiology

The age-adjusted prevalence of diabetes mellitus in adults rose from 4.7% in 1980 to 8.5% in 2014, with the greatest rise in low- and middle-income countries [21]. Based on estimates by the International Diabetes Federation for 2021, 537 million adults (aged 20–79 years) are living with diabetes mellitus [22]. This number is predicted to rise to 643 million by 2030 and to 783 million by 2045 [22]. Increasing numbers are a result of aging and increased prevalence of overweight and obesity [21,23]. Type 2 diabetes mellitus accounts for between 90% and 95% of diabetes cases [21], whereas type 1 diabetes mellitus accounts for only 1–3% of diabetic cases [23]. Both type 1 and type 2 diabetes mellitus are polygenic diseases and are by definition distinguished from monogenic forms of diabetes mellitus [24]. To date, more than 150 susceptibility loci for type 2 diabetes mellitus have been identified [25,26]. Most identified genetic variants have a small effect size individually (10–20% increased risk of type 2 diabetes mellitus per risk allele), which limits their clinical utility [27].

### 2.2. Type 2 Diabetes Mellitus: A Disease of Interaction

A simple mechanistic scheme of the pathogenesis of type 2 diabetes mellitus is hindered by the complex interrelationship between insulin sensitivity and β-cell dysfunction. Instead, it is more opportune to first describe the disease in terms of interaction of its root causes. An interaction effect is the compound impact of two or more independent variables on one or more dependent variables in which their joint effect is significantly greater (or significantly less) than the sum of the parts. In other words, the independent variables combine to have a different (and multiplicative) effect, such that the effect of one independent variable on the response variable is contingent upon the level of one or more other independent variables. For the pathogenesis of type 2 diabetes mellitus, an interaction effect occurs between environmental factors in particular caloric overload, leading to overweight and obesity and genetic factors that determine β-cell function. The cumulative effect of all genetic variants constitutes the strong genetic component of type 2 diabetes [28]. Linkage studies and genome-wide association studies have demonstrated that most risk variants for type 2 diabetes mellitus act through impaired insulin secretion, implying that β-cell dysfunction rather than insulin action is genetically determined [28]. However, environmental and lifestyle factors, via obesity, account for the current dramatic increase in type 2 diabetes mellitus [28]. The genetic predisposition per se does not inevitably lead to type 2 diabetes mellitus. The development of obesity is an important factor preceding the development of insulin resistance [29], which in the presence of a genetically determined predisposition for β-cell dysfunction resulting in reduced glucose tolerance [30].

### 2.3. β-Cell Dysfunction and Insulin Sensitivity as Mutually Dependent Determinants of Hyperglycemia in Type 2 Diabetes Mellitus

The metabolic syndrome and type 2 diabetes mellitus are associated with the presence of insulin resistance and hyperinsulinemia. Insulin sensitivity is influenced by age, acute exercise, physical fitness, dietary nutrients, obesity, and body fat distribution [30]. Central adiposity, and specifically intra-abdominal fat accumulation, is a key determinant of insulin sensitivity. Under normal physiological conditions, insulin stimulates the uptake of glucose in the liver, adipose tissue, the myocardium, the skeletal muscle, the brain, and other metabolic tissues [31]. Reduced insulin signaling in these tissues may result in systemic insulin resistance and hyperglycemia secondary to the decrease in glucose uptake. Insulin resistance also underlies diminished inhibition of hepatic glucose output, which further contributes to hyperglycemia. Hyperglycemia provokes a compensatory increase in insulin production by the pancreatic β-cells and hyperinsulinemia. However, hyperglycemia in type 2 diabetes mellitus is not only induced by insulin resistance but also by β-cell dysfunction [32]. β-cell dysfunction results from inadequate glucose sensing to stimulate insulin secretion [32]. Studies of human islets have shown reduced glucose-dependent insulin secretion in type 2 diabetes mellitus even when allowance is made for the reduction in insulin content [28]. Moreover, early insulin response in the first 30 min following oral glucose ingestion is clearly reduced in subjects with type 2 diabetes mellitus [33]. By the time hyperglycemia develops, reductions in both insulin sensitivity and in β-cell function have already occurred [30]. A temporal relationship implying that reduced insulin sensitivity precedes the occurrence of β-cell dysfunction or vice versa cannot be postulated.

The basic law that is governing the mutual effects of insulin sensitivity and β-cell function (insulin response) is that the product of insulin sensitivity and insulin response is a constant for a given degree of glucose tolerance [34]. For glucose tolerance to remain constant with varying degrees of insulin sensitivity that are experimentally manipulated, a proportionate and reciprocal alteration in insulin output must occur. Insulin sensitivity is, therefore, a modulator of insulin release and vice versa. Insulin-resistant individuals have greater insulin responses to glucose and non-glucose secretagogues, whereas insulin-sensitive subjects have smaller insulin responses. However, the magnitude of the insulin response is dictated by the degree of glucose tolerance in an individual. 

The reciprocal relationship implies that a switch of the homeostatic set points to different values of glucose tolerance occurs. Chronic inflammation and oxidative stress appear to induce a switch of the metabolic homeostatic set points [35,36]. This switch of the homeostatic set points to different values may be a strategy to deal better with the extreme or persistent abnormal conditions [35].

### 2.4. Reactive Oxygen Species and Homeostasis in Type 2 Diabetes Mellitus

Dysregulated redox homeostasis is a common pathophysiological condition, denoted as the transition from oxidative eustress to oxidative distress [36,37]. This transition may play a role in the switch of the homeostatic set points to different values of glucose tolerance. Increased reactive oxygen species decrease peripheral tissue insulin sensitivity and damage pancreatic β-cells [38]. Experimental and clinical data are consistent with an inverse association between reactive oxygen species levels and insulin sensitivity [39,40]. Serine^307^ phosphorylation of insulin receptor substrate-1 (IRS1) and increased degradation of IRS1 by a proteasome-independent pathway are induced by oxidative stress, but these changes do not strongly correlate with the oxidative stress-induced impairment of metabolic responses to insulin [40,41]. In general, oxidants have various inputs into regulating insulin signaling to the extent that type 2 diabetes is being considered as a “redox disease” [36,42]. Multiple targets and regulators of insulin receptor signaling are redox-sensitive, such as protein kinase B (Akt), forkhead box protein O (FOXO), phosphatase and tensin homolog (PTEN), protein-tyrosine phosphatase 1B (PTP1B), and c-Jun N-terminal kinases (JNKs), and, therefore, constitute plenty of nodes for redox regulation of insulin-derived signals [36,43]. High levels of oxidants favor JNK activation, which is the most studied effector of insulin resistance [44]. Oxidative distress can induce apoptosis of pancreatic β-cells, leading to decreased insulin secretion [36]. β-cells are highly vulnerable to the detrimental effects of excessive reactive oxygen species because of the lower levels of antioxidant enzymes in these cells in comparison to other tissues [45].

### 2.5. Hyperglycemia and Oxidative Stress

Adding to the complexity of the pathogenesis of type 2 diabetes mellitus is that the primary metabolic variable under consideration and the variable used to define diabetes mellitus, glucose, is itself harmful in subjects with type 2 diabetes mellitus, which is known as glucose toxicity or glucose desensitization [30]. Chronic hyperglycemia leads to the generation of oxidative stress in pancreatic β-cells [45]. Glucose lowering in these type 2 diabetic subjects leads to improved insulin sensitivity and results in improved insulin release [46]. However, this glucose toxicity is not observed in healthy subjects in which a continuous infusion of glucose induces enhanced insulin sensitivity and β-cell function [47]. Hyperglycemia promotes overproduction of reactive oxygen species by the mitochondrial electron transport chain [38,48] and exacerbates formation of advanced glycation end-products (AGE). Normalizing levels of mitochondrial reactive oxygen species induced by hyperglycemia in cultured bovine aortic endothelial cells prevent glucose-induced activation of protein kinase C, formation of AGE, sorbitol accumulation, and nuclear factor kappa B (NF-κB) activation [49]. The interaction between AGE and the receptor for AGE (RAGE) stimulates NADPH oxidase-1 which contributes to reactive oxygen species production in diabetes [50,51]. The interactions of AGE and their receptor RAGE also lead to the upregulation of NF-κB [52]. Sustained activation of NF-κB induces the systemic inflammation, one of the features of chronic diabetes.

### 2.6. Homeostatic Setpoints and Glucose Tolerance 

The development of glucose intolerance and diabetes mellitus reflects a reset of homeostatic set points. Chronic inflammation and oxidative stress appear to induce a switch of the metabolic homeostatic set points [35,36]. However, the preceding paragraph might result in a chicken or egg discussion. Not all subjects with obesity or insulin resistance develop glucose intolerance. Early impairment of β-cell function based on a genetic predisposition may, via increased glucose output, reduced efficiency of glucose uptake, and increased lipolysis, result in mildly increased glucose levels and non-esterified fatty acid (NEFA) levels [53] that via a feedforward mechanism produce a vicious cycle that begets more insulin resistance and β-cell dysfunction. High glucose and NEFA promote production of interleukin-1β in the islets of Langerhans, which may elicit cytokine-induced β-cell apoptosis [54,55,56]. Further detrimental effects of hyperglycemia have been described in the previous paragraph. Increased NEFA levels have deleterious effects both on insulin-sensitive tissues and on the β-cells [30,53]. Exposure to NEFA induces changes in post-receptor insulin signaling, decreases insulin-mediated glucose uptake and glycogen synthesis, and reduces β-cell function [57,58,59]. Chronically elevated blood levels of NEFA lead to lipotoxicity in many cell types including β-cells, leading to a decrease in β-cell functionality and viability [60].

## 3. Oxidative Stress and Diabetic Cardiomyopathy

### 3.1. The Myocardium as a Predilection Site of Oxidative Stress

Under physiological conditions, there is a delicate balance between reactive oxygen species production and reactive oxygen species degradation resulting in functional steady-state reactive oxygen species levels [9]. Redox-sensitive pathways are indispensable for normal cardiac physiology and homeostasis, and low levels of reactive oxygen species are involved in modulation of excitation–contraction coupling, physiological hypertrophy, and cardiac homeostasis [9,61]. In contrast, oxidative stress in the heart secondary to a transient or persistent increment in steady-state reactive oxygen species levels results in disturbed signaling pathways and oxidative modification of cellular components, which subsequently can lead to cell dysfunction and even induce cell death via necrosis or apoptosis [9,62]. Protein oxidation, lipid peroxidation, DNA damage, and oxidative changes in microRNAs contribute to cellular dysfunction [63,64]. Moreover, reactive oxygen species can activate a multitude of cellular stress-sensitive pathways that include JNKs, extracellular signal-regulated kinase 1 and 2, p38 mitogen-activated protein kinases, NF-κB, and protein kinase C [65,66,67]. High levels of reactive oxygen species and/or more potent oxidants such as HO^.^ result in the activation of pathological processes such as impaired calcium handling, cardiomyocyte hypertrophy, pronounced interstitial and perivascular myocardial fibrosis, and myocardial apoptosis [9,12,61]. Apoptosis has been shown to be markedly increased in the myocardium of human diabetic subjects [68].

Oxidative stress is also closely linked to endoplasmic reticulum stress and the unfolded protein response. Since the protein folding process is dependent on redox homeostasis, oxidative stress may disrupt the protein folding mechanism and enhance the production of misfolded proteins [69]. In addition, lipotoxicity and inflammation also impair the function of the cardiac endoplasmic reticulum, leading to endoplasmic reticulum stress and the unfolded protein response [70,71]. Homeostatic activation of the unfolded protein response enforces adaptive programs that modulate and augment key aspects of the entire secretory pathway, whereas a maladaptive unfolded protein response occurring under conditions of high intensity and long duration of endoplasmic reticulum stress triggers apoptosis [72].

Obesity, the metabolic syndrome, and diabetes mellitus are marked by chronic low-grade inflammation and a state of permanently increased oxidative stress [9,73]. Biomarkers of chronic inflammation are increased in type 2 diabetes mellitus as evidenced by increased concentrations of C-reactive protein [74,75]. Similarly, biomarkers of chronic oxidative stress are augmented as evidenced by increased levels malondialdehyde, thiobarbituric acid reactive substances (TBARS), hydroxynonenal (HNE), 8-hydroxyguanosine, 8-hydroxy-2′-deoxyguanosine, and 8-iso-prostaglandin F_2α_ [75]. The heart is extremely susceptible to oxidative distress. After all, the heart is metabolically the most active organ and is characterized by the highest density of mitochondria of any tissue. Mitochondria encompass 25–30% of cell volume across different mammalian species [76,77]. The mitochondrial respiratory chain contains approximately 92 nuclear and mitochondrial DNA-encoded protein subunits that are organized into five different multi-subunit respiratory complexes [78]. Between 0.2% and 2% of electrons may leak from the respiratory chain and react with O_2_, leading to the production of superoxide (O_2_^−^) during oxidative phosphorylation [63]. This process of electron leakage and O_2_^−^ formation is of particular importance in the diabetic heart. Other sources of reactive oxygen species are NADPH oxidases, xanthine oxidase, cytochrome P450 enzymes, and uncoupling of nitric oxide synthase [9,63,79,80].

Hyperglycemia promotes overproduction of reactive oxygen species by the mitochondrial electron transport chain [38,48] and contributes to oxidative distress. Four main molecular mechanisms have been implicated in glucose-mediated damage: increased polyol pathway flux, increased AGE formation and activation of RAGE, activation of protein kinase C isoforms, and increased hexosamine pathway flux [48]. All four mechanisms are activated by a single upstream event: mitochondrial overproduction of reactive oxygen species [48,81]. Diabetes in animals and patients decreases the activity of the key glycolytic enzyme glyceraldehyde 3-phosphate dehydrogenase in cell types that develop intracellular hyperglycemia [81]. When glyceraldehyde 3-phosphate dehydrogenase activity is inhibited, this results in an increase in the level of all of the glycolytic intermediates that are upstream of this enzyme, resulting in an elevated flux into the four pathways mentioned supra [81]. Inhibition of glyceraldehyde 3-phosphate dehydrogenase activity by hyperglycemia does not occur when mitochondrial overproduction of superoxide is prevented by either uncoupling protein 1 or the mitochondrial tetrameric manganese superoxide dismutase (SOD2) [82], indicating the key role of mitochondrial superoxide. Glyceraldehyde 3-phosphate dehydrogenase inhibition was observed to be the consequence of poly(ADP-ribosyl)ation by poly(ADP-ribose) polymerase (PARP), which was activated by DNA strand breaks produced by mitochondrial superoxide overproduction [82]. 

**Figure 1:**
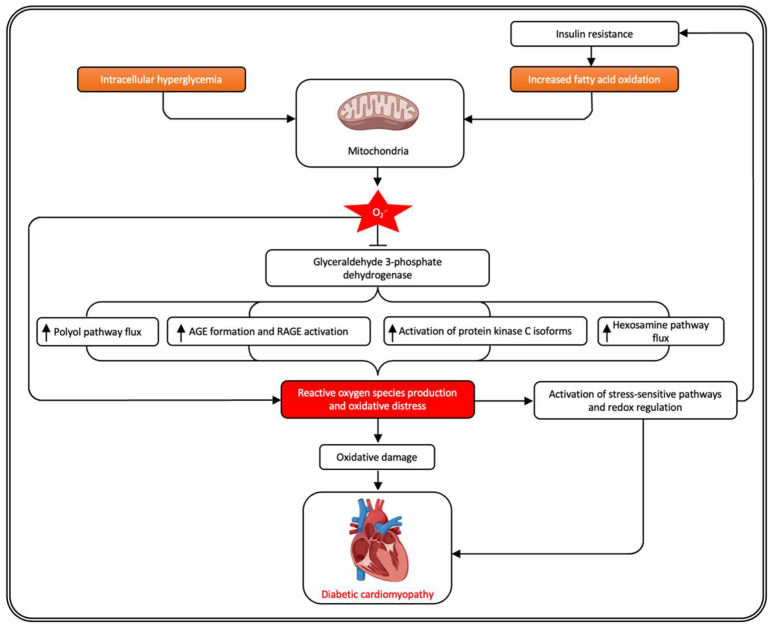
Central role of reactive oxygen species and oxidative distress in the development of diabetic cardiomyopathy. Mitochondrial overproduction of superoxide anion radicals induces, via inhibition of glyceraldehyde 3-phosphate dehydrogenase, an increased polyol pathway flux, increased advanced glycation end-products (AGE) formation and activation of the receptor for AGE (RAGE), activation of protein kinase C isoforms, and an increased hexosamine pathway flux. These pathways not only directly contribute to diabetic cardiomyopathy (arrows not shown) but are themselves a source of additional reactive oxygen species and oxidative distress. Oxidative distress itself is also a cause of insulin resistance.

Intracellular hyperglycemia is observed in the diabetic microvasculature [81]. Endothelial cells constitute the predominant cell type in the myocardium as described by Pinto et al. [83]. These investigators showed, based on immunohistochemical analysis of human cardiac tissue, that 31% of nuclei in the human heart correspond to cardiomyocytes (α-actinin 2 positive cells), 54% to endothelial cells (CD31 positive cells), 3% to leukocytes (CD45 positive cells), and the remaining to resident mesenchymal cells including fibroblasts [83]. In contrast to the diabetic microvasculature, mitochondrial overproduction of reactive oxygen species in the diabetic macrovasculature and in the heart is caused by increased oxidation of fatty acids, resulting in part from pathway-specific insulin resistance [81]. Glycogen synthase kinase-3β plays a critical role in cardiac glucose metabolism. As discussed in Section 2, reactive oxygen species contribute to compromised insulin signaling. Impaired insulin signaling in the cardiomyocytes of both type 1 and type 2 diabetic patients depresses Akt-dependent glycogen synthase kinase-3β phosphorylation (inactivation) [84], which alters energy metabolism. The diabetic heart is characterized by diminished glucose utilization and increased fatty acid oxidation, which may result in cardiac lipid accumulation [85,86]. Myocardial lipotoxicity may be a major trigger for cardiac inflammation and oxidative damage [85,86]. Cardiac lipotoxicity, characterized by increased uptake, oxidation, and accumulation of lipid intermediates, is accompanied by mitochondrial dysfunction [87]. Using transgenic mice overexpressing long-chain acyl-CoA synthetase 1 in cardiomyocytes, a model of cardiac lipotoxicity, it was demonstrated that increased myocardial fatty acid uptake led to mitochondrial structural remodeling with significant reduction in minimum diameter and was associated with increased palmitoyl-carnitine oxidation and increased reactive oxygen species generation in isolated mitochondria [87]. Therefore, mitochondrial overproduction of reactive oxygen species is not only caused by intracellular hyperglycemia [81] but is also the result of increased fatty oxidation and lipotoxicity in cardiomyocytes [87] (Figure 1). Above all, free fatty acid-induced reactive oxygen species overproduction has been shown to have effects similar to those of glucose-induced reactive oxygen species on glyceraldehyde 3-phosphate dehydrogenase activity and downstream effects on the hexosamine pathway activity, protein kinase C activity, and AGE formation [88]. Moreover, the Randle cycle, also known as the glucose fatty-acid cycle, describes the reciprocal relationship between fatty acid and glucose metabolism. Consequently, there is a profound direct impact of fatty acid oxidation on glycolysis and on the activity of pyruvate dehydrogenase [85].

As will be discussed in the following sections, the downstream effects of the four pathways initiated by diabetes and the mitochondrial overproduction of reactive oxygen species [81] may themselves also exacerbate oxidative stress (Figure 1). Taken together, the effects of oxidative stress in the diabetic heart are not only mediated by direct damage of proteins, lipids, DNA, and microRNAs and by effects on redox-sensitive signaling [65] but also by downstream effects of the pathways (polyol pathway, AGE formation and RAGE activation, activation of protein kinase C isoforms, and increased hexosamine pathway flux) that are typically set in motion in diabetes (Figure 1).

### 3.2. The Polyol Pathway, Oxidative Stress, and Diabetic Cardiomyopathy 

High levels of glucose are metabolized through aldose reductase and sorbitol dehydrogenase to generate high intracellular levels of polyols and fructose [89]. Aldose reductase reduces glucose to sorbitol in the presence of NADPH, and sorbitol dehydrogenase oxidizes sorbitol to fructose using NAD^+^. Excessive consumption of NADPH due to increased activity of aldose reductase in the polyol pathway invariably decreases the level of intracellular glutathione (GSH), an endogenous free radical scavenging antioxidant in its active form [90]. Elevation of the cytosolic NADH/NAD^+^ ratio leads to excess production of reactive oxygen species including superoxide (O_2_^−^) via cytosolic and mitochondrial NADH-dependent pathways [89]. Interestingly, cardiomyocyte-specific expression of human aldose reductase under control of the α–myosin heavy chain promoter caused cardiac dysfunction in older transgenic mice [91]. AT-001 is a novel aldose reductase inhibitor, which is currently being evaluated in adult patients with diabetic cardiomyopathy at high risk of progression to overt heart failure (NCT04083339) [92]. AT-001 treatment for 28 days has been demonstrated to decrease blood levels of sorbitol and N-terminal pro-B-type natriuretic peptide levels in diabetic patients [92].

### 3.3. AGE, RAGE, Oxidative Stress, and Diabetic Cardiomyopathy

Glycation is the non-enzymatic reaction between reducing sugars, such as glucose, and proteins, lipids or nucleic acids [93]. During the classical Maillard reaction, electrophilic carbonyl groups of a reducing sugar react with free amino groups of proteins (especially of basic lysine or arginine residues) to form a reversible Schiff base. Through rearrangement, a more stable Amadori product (ketoamine) is formed. Schiff bases and Amadori products are reversible reaction products but can react irreversibly with amino acid residues of peptides or proteins to form protein adducts or protein crosslinks [93]. Advanced glycation end-products (AGE) is the collective name given to proteins, lipids, and nucleic acids that undergo irreversible modification by reducing sugars or sugar-derived products. Formation of AGE is a complicated molecular process involving multistep reactions [93]. Most AGE are formed by a combination of glycation and oxidation reactions and are termed glycoxidation products [93]. AGE can be formed under physiological conditions, but their formation is potently augmented under conditions of hyperglycemia. AGE are prominently present in the diabetic heart and may play an important role in the pathogenesis of diabetic cardiomyopathy [94]. RAGE is a member of the immunoglobulin superfamily of cell surface molecules. It is a pattern recognition receptor, and the binding of ligands to RAGE stimulates various signaling pathways [95]. The interaction between AGE and RAGE stimulates NADPH oxidase-1 which contributes to reactive oxygen species production in diabetes [50,51]. Stimulation of RAGE also results in activation of the transcription factor NF-κB and subsequent transcription of several proinflammatory genes [95]. In addition, NF-κB induces expression of RAGE, which itself further stimulates NF-κB activity, forming a vicious cycle of self-renewing and perpetuating proinflammatory signals [95,96,97]. The development of diabetic cardiomyopathy was partially counteracted by application of in vivo RAGE gene knockdown using RNA interference in mice with streptozotocin-induced diabetes [98]. Blocking RAGE with a RAGE antibody also prevented diabetic cardiomyopathy in db/db diabetic mice [99]. Interestingly, soluble levels of RAGE can be measured in serum or plasma. A correlation has been observed between soluble levels of RAGE and severity of heart failure, expressed as New York Heart Association heart failure class [100,101]. Non-enzymatic glycation may also alter the structure and function of antioxidant enzymes to the extent that they are unable to detoxify free radicals, exacerbating oxidative distress in diabetes [102,103].

### 3.4. Protein Kinase C β, Oxidative Stress, and Diabetic Cardiomyopathy

The gradual impairment of insulin secretion and insulin signaling in diabetes is associated with elevated NEFA and increased myocardial free fatty acid uptake, accumulation of diacylglycerol, and protein kinase C activation [104]. Oxidative stress induces protein kinase Cβ activation, leading to p66^Shc^ phosphorylation, which allows its recognition by peptidyl-prolyl cis/trans isomerase (Pin-1) and its subsequent transfer from the cytosol to the mitochondrion [105]. In the mitochondrion, p66^Shc^ induces permeability transition pore opening, triggering its mitochondrial proapoptotic effects. The protein p66^Shc^ induces mitochondrial H_2_O_2_ production and so further increases intracellular H_2_O_2_ levels. Therefore, p66^Shc^ can maintain or increase protein kinase Cβ activation in a kind of self-triggered control loop [106]. Interestingly, mice knocked out for the gene encoding the p66^shc^ adaptor protein are resistant to oxidative stress and have an increased life span [107]. Moreover, absence of the p66^shc^ adaptor protein protected against the development of diabetic cardiomyopathy in a model of streptozotocin-induced diabetes mellitus [108]. 

Protein kinase Cβ2 is overexpressed in the diabetic myocardium, which contributes to cardiomyocyte hypertrophy, to myocardial fibrosis, and to the development of diabetic cardiomyopathy [109,110,111]. Prevention of excessive protein kinase Cβ2 activation attenuated cardiac diastolic dysfunction by restoring caveolin-3 expression and subsequently rescuing Akt/endothelial nitric oxide synthase (eNOS)/nitric oxide signaling [112]. Protein kinase Cβ inhibition with ruboxistaurin reduced oxidative stress and attenuated left ventricular hypertrophy and dysfunction in rats [110]. Taken together, these findings indicate myocardial protein kinase Cβ is a major contributor of oxidative stress in the diabetic heart [110].

### 3.5. Hexosamine Pathway, Oxidative Stress, and Diabetic Cardiomyopathy 

Hyperglycemia and insulin resistance-induced excess fatty acid oxidation, which occurs in the myocardium, contribute to the pathogenesis of diabetic complications by increasing the flux of fructose 6-phosphate into the hexosamine pathway [113]. Hexosamine biosynthesis normally is only a minor alternative metabolic pathway for glucose carbon at the fructose-6-phosphate step of glycolysis [114]. However, hexosamine biosynthetic pathway flux augments under conditions when glucose is imported into cardiomyocytes in excess of their capacity to readily metabolize it via glycolysis and pyruvate oxidation or under conditions of overload of free fatty acids whose uptake and metabolism inhibit pyruvate oxidation [114]. As already stated, oxidative stress may also augment hexosamine biosynthetic pathway flux by inhibiting glyceraldehyde-3-phosphate dehydrogenase, the rate-limiting enzyme of glycolysis [113]. The major product of the hexosamine biosynthetic pathway, UDP-N-acetylglucosamine (UDP-GlcNAc), is the required substrate for O-GlcNAc transferase (OGT), the enzyme that catalyzes the reversible O-GlcNAcylation of specific serine/threonine residues of several cytosolic and nuclear proteins. This posttranslational O-GlcNAcylation interferes with phosphorylation of these same serine/threonine residues, leading to a change in their function. Some of the key changes involve eNOS and sarcoendoplasmic reticulum Ca^2+^-ATPase 2a (SERCA2a). The activity of eNOS in endothelial cells is inhibited by O-GlcNAcylation at the Akt activation site of the eNOS protein [115]. Increased nuclear O-GlcNAcylation decreases SERCA2a promoter activity, leading to reduced SERCA2a mRNA and protein expression levels [116]. The process of O-GlcNAcylation has a significant impact on cardiac excitation–contraction coupling and cardiac function [116,117]. The transition from normoglycemia to hyperglycemia in Zucker diabetic fatty rats, a model of type 2 diabetes mellitus, is associated with intracellular accumulation of UDP-GlcNAc and O-GlcNAc-modified proteins, delayed calcium sequestration, and impaired mechanical relaxation [118]. Activation of the hexosamine pathway may cause oxidative stress through depletion of GSH [119,120]. A link between hyperglycemia, oxidative stress, and the hexosamine biosynthetic pathway leading to cardiomyocyte apoptosis has been demonstrated in cardiomyoblasts [121].

### 3.6. MicroRNAs and Oxidative Stress in Diabetic Cardiomyopathy

A complex crosstalk exists between microRNAs and reactive oxygen species in diabetic cardiomyopathy [122]. On the one hand, microRNAs may at the post-transcriptional level regulate expression of genes that contribute to oxidative stress or attenuate oxidative stress. Conversely, oxidative stress regulates microRNAs that control genes involved in mechanisms that mitigate damage induced by reactive oxygen species [122]. This topic is outside the scope of the current manuscript, and we refer to several recent reviews specifically dedicated to this subject [123,124,125,126].

## 4. Prevention and Intervention Studies Directly Supporting the Role of Oxidative Stress in Diabetic Cardiomyopathy

### 4.1. Primary Antioxidant Enzymes

**Figure 2:**
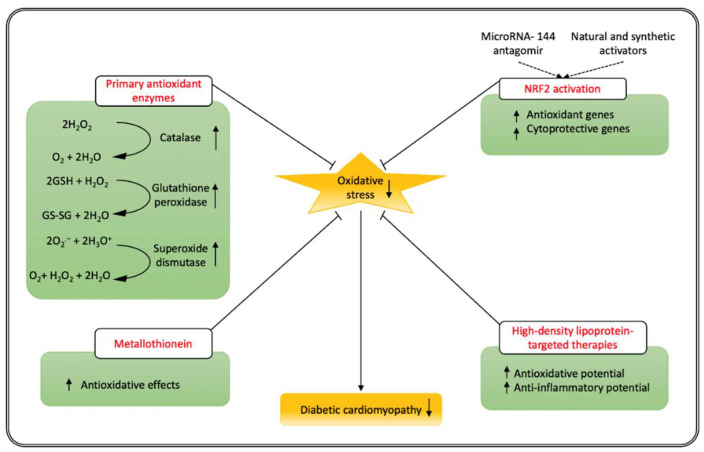
Prevention and intervention studies directly supporting the role of oxidative stress in diabetic cardiomyopathy. Antioxidant strategies prevent the development of diabetic cardiomyopathy, supporting the central role of oxidative stress in the pathogenesis of this disorder. HDL-targeted therapies, increasing the anti-oxidative potential of HDL, not only prevent diabetic cardiomyopathy but also result in reverse remodeling and reversal of heart failure in pre-existing diabetic cardiomyopathy.

Studies in transgenic animals expressing the primary antioxidant enzymes are the most direct and specific tool to demonstrate, in a prevention setting, the causal role of oxidative stress in diabetic cardiomyopathy [9]. The three primary antioxidant enzymes are superoxide dismutase, catalase, and glutathione peroxidase. Superoxide dismutases (SODs) are a family of isoenzymes involved in the scavenging of O_2_^−^ and catalyze the same reaction converting O_2_^−^ in O_2_ and H_2_O_2_ [127]. The enzymes catalase and glutathione peroxidase degrade H_2_O_2_ (Figure 2). In the diabetic heart, overexpression of the mitochondrial tetrameric manganese superoxide dismutase (SOD2) or catalase protects cardiac mitochondria from oxidative damage, improves respiration, and normalizes mass in diabetic mitochondria [128,129]. SOD2 also prevents the morphological changes in diabetic hearts and completely normalizes contractility in diabetic cardiomyocytes. [128]. Overexpression of glutathione peroxidase under control of a housekeeping gene promoter prevented the development of diabetic cardiomyopathy in mice with streptozotocin-induced diabetes mellitus [130]. An improvement of left ventricular diastolic function, an attenuation of cardiomyocyte hypertrophy, a decrease in interstitial fibrosis, and a reduction in myocardial apoptosis were all induced by overexpression of glutathione peroxidase [130].

Unfortunately, intervention studies applying gene transfer of the primary antioxidant enzymes, evaluating their effect on established diabetic cardiomyopathy, have not been performed. This issue should be addressed in future studies.

### 4.2. Metallothionein

Metallothioneins are low molecular weight, cysteine-rich, metal-binding proteins that are involved in heavy metal detoxification, metal ion homeostasis, pancreatic β-cell biology, and antioxidant defense [131]. Metallothionein expression under control of the cardiomyocyte-specific mouse cardiac α-myosin heavy chain promoter in the heart of transgenic mice [132] protected against the development of diabetic cardiomyopathy in a model of type 1 diabetes mellitus and in a model of streptozotocin-induced diabetes mellitus [133,134]. Although the effect of metallothionein on diabetic cardiomyopathy has been attributed to its potent antioxidative effects [134,135], several other mechanisms may play a role [136,137]. In particular, activation (dephosphorylation) of glycogen synthase kinase-3β, which is a key enzyme in the regulation of metabolism, is observed in the hearts of wild-type diabetic mice but not in metallothionein-overexpressing transgenic mice [137]. However, the root cause of the beneficial effects of metallothionein is likely related to its antioxidant properties (Figure 2). 

### 4.3. Reduction of Oxidative Stress via Enhanced Activity of the Transcription Factor Nuclear Factor-Erythroid 2 p45-Related Factor 2 (NRF2) 

The transcription factor nuclear factor-erythroid 2 p45-related factor 2 (NRF2) is activated in response to increased H_2_O_2_ levels [138]. NRF2 binds to the antioxidant responsive element (ARE) [139] and induces the expression of multiple antioxidant genes (superoxide dismutase, catalase, glutathione peroxidase-1, glutathione synthesis) and cytoprotective genes (heme oxygenase-1, NAD(P)H quinone dehydrogenase 1) [140]. It plays a crucial role in cardiovascular redox homeostasis and suppresses oxidative stress [139]. NRF2 confers protection against high glucose-induced oxidative damage in cardiomyocytes [141]. Under physiological conditions, NRF2 binds Kelch-like ECH-associated protein 1 (KEAP1) and the Cullin 3 (CUL3)-based E3 ubiquitin ligase [142,143]. Following stimulation by reactive oxygen species, the NRF2-KEAP1 complex is disrupted and NRF2 translocates to the nucleus, where it interacts with the ARE, resulting in the transcription of NRF2-dependent antioxidant genes [142,143]. MicroRNA-144 directly recognizes the 3′-UTR of NRF2 and represses NRF2 expression [144]. Treatment with microRNA-144 antagomir (Figure 2) in mice with streptozotocin-induced diabetic cardiomyopathy up-regulated NRF2 protein expression, suppressed reactive oxygen species formation, and decreased malondialdehyde content [145]. At the cellular level, treatment with microRNA-144 antagomir initiated seven days after the induction of diabetes mellitus reduced caspase-3 activation and terminal deoxynucleotidyl transferase dUTP nick end labeling (TUNEL)-positive cells in the left ventricular myocardium, indicating reduced myocardial apoptosis. Finally, microRNA-144 antagomir treatment improved cardiac function in mice with streptozotocin-induced diabetes mellitus [145].

Natural activators of NRF2 are sulforaphane, curcumin, quercetin, xanthohumol, sulforaphane, and resveratrol [146], whereas synthetic activators of NRF2 include bardoxolone-methyl, omaveloxolone, dimethyl fumarate, and oltipraz [147]. Activating NRF2 results in pleiotropic effects since NRF2 turns on the transcription of more than 200 genes in the human genome [66]. Many natural and synthetic activators of NRF2 have been demonstrated to be efficacious in preventing diabetic cardiomyopathy in animal models [66]. However, safety issues should be considered since NRF2 is overexpressed in many human cancers including lung, breast, ovarian, head and neck, and endometrial cancers in humans [66]. More importantly, NRF2 activation by antioxidant antidiabetic agents accelerates tumor metastasis of existing tumors [148]. This safety dimension should be considered for other strategies whose immediate or downstream effects include NRF2 activation.

### 4.4. High-Density Lipoprotein-Targeted Therapies: The Construction of Antioxidant Multimolecular Platforms 

High-density lipoproteins (HDL) are circulating multimolecular platforms that can exert multiple tasks with a prominent role for the antioxidative anti-inflammatory functions of these particles [149,150,151,152] (Figure 2). Biochemical heterogeneity of HDL particles [153], which mainly contain apolipoprotein (apo) A-I, cholesterol, and phospholipids, is based on the mutable presence of one or more representatives of at least 250 proteins, 300 lipid species, and 20 micro RNAs [154]. Important contributors to the antioxidative and anti-inflammatory potential of HDL are paraoxonase or platelet activating factor-acetyl hydrolase, 2 enzymes associated with HDL that are increased under conditions of augmented *apo A-I* levels induced by HDL-targeted therapies [151,155]. Human *apo A-I* gene transfer reduced oxidative stress in diabetes via a decrease in NADPH oxidase activity and by counteracting eNOS uncoupling [156]. Human *apo A-I* gene transfer reduced oxidative stress in rats with streptozotocin-induced diabetes as indicated by the decrease in serum levels of thiobarbituric acid reactive substances (TBARS) [157], a generally used metric of lipid peroxidation in biological samples. In addition, the activated phosphorylation state of the stress-induced p38 mitogen-activated protein kinase was reduced by HDL-raising gene therapy in animals with streptozotocin-induced diabetes mellitus [157]. Moreover, glycogen synthase kinase-3β phosphorylation (inactivation) was increased following human *apo A-I* gene transfer in diabetic rats [157]. Pre-emptive human *apo A-I* gene transfer prevented the development of diabetic cardiomyopathy in this model as evidenced by improved cardiac function and by reduction of myocardial apoptosis, myocardial inflammation, and myocardial fibrosis [157].

More strikingly, reversal of diabetic cardiomyopathy following an intervention with apo A-I_Milano_ nanoparticles was demonstrated in a type 2 diabetes model of cardiomyopathy induced by feeding a high-sugar/high-fat (HSHF) diet between the age of 12 and 28 weeks in C57BL/6N mice [158]. This HSHF diet induced obesity, hyperinsulinemia, type 2 diabetes mellitus, and diabetic cardiomyopathy. The induction of diabetic cardiomyopathy by the HSHF diet in mice was evidenced by the presence of cardiac hypertrophy, prominent interstitial and perivascular fibrosis, decreased myocardial capillary density, systolic and diastolic dysfunction, and heart failure. Treatment with apo A-I_Milano_ nanoparticles initiated at the age of 28 weeks reversed pathological remodeling and cardiac dysfunction and normalized wet lung weight, indicating effective treatment of heart failure [158]. This reverse remodeling occurred in the presence of a reduction in nitro-oxidative stress in the myocardium and in the presence of a pronounced decrease in inflammation [158].

### 4.5. Curcumin

Curcumin is a natural polyphenolic compound that was originally used in traditional Indian medicine over 3000 years ago and is isolated from turmeric (*Curcuma longa*). This compound possesses diverse pharmacological properties including antioxidant activity [159]. Curcumin activates the NRF2 system [160]. Curcumin attenuated the development of diabetic cardiomyopathy in rats with streptozotocin-induced diabetes by attenuating oxidative stress and myocardial inflammation, which resulted in decreased myocardial fibrosis and reduced cell death and improved cardiac function [161]. The protective effect of curcumin on diabetic cardiomyopathy is associated with increased heme oxygenase-1 expression, catalase expression, superoxide dismutase expression, and glutathione levels, and reduced expression of the NAD(P)H oxidase subunits p22 phox, p47 phox, p67 phox, and gp91 phox [66,161,162,163]. Curcumin has been evaluated quite extensively in clinical trials in subjects with diabetes mellitus [164]. These studies have demonstrated favorable outcomes on biomarkers, but effects on hard clinical endpoints have not been demonstrated until now [164].

## 5. Conclusions

Since the heart is metabolically the most active organ and is characterized by the highest content of mitochondria of any tissue, it is extremely susceptible to oxidative distress. Oxidative stress in the heart secondary to transient or persistent increase in steady-state reactive oxygen species levels results in disturbed signaling pathways and oxidative modification of cellular constituents, which subsequently can induce cell dysfunction or even cell death via necrosis or apoptosis [9,62]. Mitochondrial overproduction of reactive oxygen species is the trigger for damage induced by increased polyol pathway flux, increased AGE formation and activation of RAGE, activation of protein kinase C (PKC) isoforms, and increased hexosamine pathway flux [48]. These pathways are a source of increased reactive oxygen species production, perpetuating oxidative stress.

As oxidative stress is related to a multitude of pathways in diabetes mellitus, it might seem very difficult to prove its causal role in diabetic cardiomyopathy. Nevertheless, investigations in animal models of diabetic cardiomyopathy have consistently demonstrated that increased expression of the primary antioxidant enzymes attenuates myocardial pathology and improves cardiac function, indicating that oxidative stress is not simply a bystander but a key player in diabetic cardiomyopathy. This thesis is further supported by the beneficial effects of other anti-oxidative strategies. Interestingly, HDL-targeted therapies that increase the anti-oxidative potential of HDL not only prevent diabetic cardiomyopathy but also result in reverse remodeling and reversal of heart failure in established diabetic cardiomyopathy [158]. However, whereas there is a plenitude of prevention studies evaluating the impact of antioxidant strategies on the development of diabetic cardiomyopathy, there is a paucity of experimental intervention studies that investigate the effect of these strategies on existing diabetic cardiomyopathy and heart failure. A key question is whether gene transfer of the primary antioxidant enzymes constitutes a successful therapy to reverse diabetic cardiomyopathy. These experiments may constitute the ultimate proof that oxidative stress is a key player in diabetic cardiomyopathy.
